# Transcriptional Control by Premature Termination: A Forgotten Mechanism

**DOI:** 10.1016/j.tig.2019.05.005

**Published:** 2019-08

**Authors:** Kinga Kamieniarz-Gdula, Nick J. Proudfoot

**Affiliations:** 1 Sir William Dunn School of Pathology, University of Oxford, South Parks Road, Oxford, OX1 3RE, UK; 2 Department of Molecular and Cellular Biology, Institute of Molecular Biology and Biotechnology, Faculty of Biology, Adam Mickiewicz University, Umultowska 89, 61-614 Poznań, Poland; 3 Center for Advanced Technology, Adam Mickiewicz University, Umultowska 89c, 61-614 Poznań, Poland

**Keywords:** premature transcription termination, transcription attenuation, alternative polyadenylation (APA), intronic polyadenylation (IPA), alternative last exon (ALE)

## Abstract

The concept of early termination as an important means of transcriptional control has long been established. Even so, its role in metazoan gene expression is underappreciated. Recent technological advances provide novel insights into premature transcription termination (PTT). This process is frequent, widespread, and can occur close to the transcription start site (TSS), or within the gene body. Stable prematurely terminated transcripts contribute to the transcriptome as instances of alternative polyadenylation (APA). Independently of transcript stability and function, premature termination opposes the formation of full-length transcripts, thereby negatively regulating gene expression, especially of transcriptional regulators. Premature termination can be beneficial or harmful, depending on its context. As a result, multiple factors have evolved to control this process.

## Highlights

PTT is widespread in metazoans. It can occur close to the TSS or further downstream in the gene body.
PTT generates transcripts that, depending on the circumstances, are either rapidly degraded, or are stabilised by polyadenylation, thus contributing to transcriptome diversification.
Stable premature transcripts can have independent functions as noncoding (nc)RNA or mRNA encoding proteins with different properties compared with those generated by the full-length transcript.
PTT can negatively regulate expression of the full-length transcript and especially controls genes encoding transcriptional regulators.
Factors triggering PTT include not only canonical RNA 3′ processing and termination factors, but also other players. Many metazoan factors oppose PTT, thus limiting its damaging potential.

## Transcriptional Control by Premature Termination: Revisited

**Figure 1:**
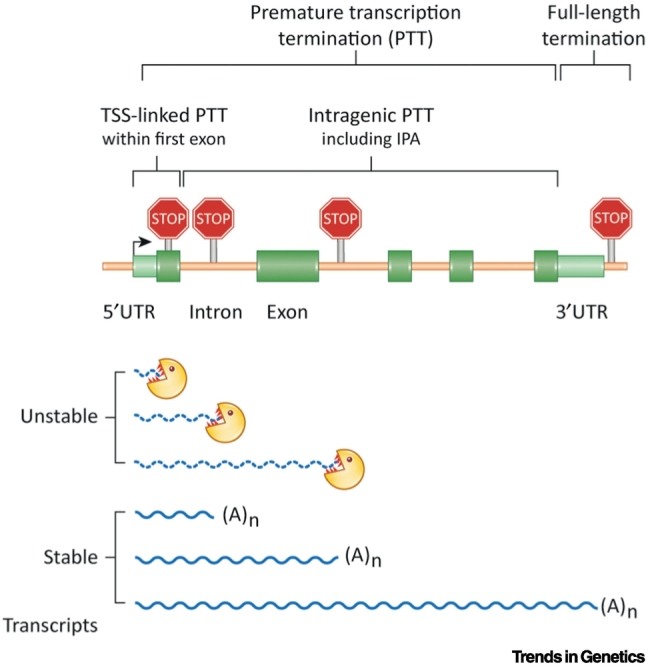
Key Figure. Localisation of Premature and Full-Length Transcription Termination. We classify any transcription termination events occurring within the 3′-untranslated region (UTR) of the full-length transcript or downstream of the annotated 3′-UTR, as full-length, whereas those occurring before as premature. Premature transcription termination (PTT) can occur in the vicinity of the transcription start site (TSS), where we call it TSS-linked PTT, or further downstream in the gene body, described here as intragenic PTT. As a rule of thumb, we annotate TSS-linked PTT as corresponding to termination events within the first exon; however, please note that this is not an absolute distinction and is likely to vary on a gene-by-gene basis. PTT can generate unstable (dotted wavy line) or stable (solid wavy line), polyadenylated transcripts. If PTT generates a stable, polyadenylated transcript, it can be classified as alternative polyadenylation (APA) or intronic polyadenylation (IPA). Abbreviation: (A)_n_, polyadenylation.

It is well established that early termination can serve as an important mechanism for transcriptional control. **PTT** (see Glossary), or ‘attenuation’, was demonstrated during the mid-1970s to be a key regulatory event for the synthesis of bacterial enzymes that make amino acids 1., 2., and first reported in 1979 to occur for **RNA polymerase II** (Pol II) transcription of a viral gene in mammalian cells [3]. Many more cases of eukaryotic PTT have been identified, even though their analysis was hampered by technical limitations and the highly unstable nature of prematurely terminated RNA. The recent development of next-generation sequencing technologies combined with novel methods to measure nascent transcription, single-molecule footprints, and advanced live-imaging makes it possible to revisit this paradigm. In this review, we present recent findings on metazoan PTT, revealing its widespread nature and role in the regulation of protein-coding genes. While we focus on metazoans, a broader perspective of PTT in other kingdoms of life is summarised in Box 1. Our definition of PTT is the release of Pol II from the gene template between the **TSS** and 3′-**untranslated region** (UTR; Figure 1, Key Figure) of the gene. We note that **transcription termination** is tightly linked with RNA 3′ **cleavage and polyadenylation** (CPA). Consequently, these two terms are often used ambiguously or even confused in the literature. Multiple recent reviews provide a general background to RNA 3′ processing and transcription termination 4., 5., 6., 7., 8., 9., 10., 11. as well as **APA** 12., 13., 14., 15., 16..

Box 1 **PTT in Bacteria, Yeast and Plants**

PTT has been long known to be a key regulatory event in bacteria, referred to as attenuation. Classically, attenuation was shown to control the expression of enzymes involved in amino acid biosynthesis, such as the tryptophan and histidine operons 1., 2.. Bacterial terminators can be intrinsic, associated with a hairpin RNA structure, or factor dependent, usually involving the RNA helicase Rho. Attenuation occurs when an antiterminator hairpin RNA forms ahead of an intrinsic terminator positioned near the 5′ end of an operon. Formation of the antiterminator hairpin precludes the formation of the intrinsic terminator hairpin and so allows transcription to read into the operon and express its protein-coding regions. Switching between the antiterminator and terminator hairpins is controlled by diverse regulators 100., 101.. Given that translation occurs co-transcriptionally, PTT is closely coupled to translation regulation. This differentiates it from eukaryotic regulation.

PTT is also a well-recognised regulatory mechanism in *Saccharomyces cerevisiae*, mediated by the Nrd1–Nab3–Sen1 (NNS) complex. The first example of attenuation by NNS was demonstrated for the *NRD1* gene, which is autoregulated by PTT in response to Nrd1 activity [98]. NNS-mediated PTT further regulates genes involved in nucleotide and amino acid biosynthesis, as well as nitrogen metabolism, and is physiologically relevant upon nutritional shift 102., 103., 104., 105.. The prematurely terminated transcripts sometimes initiate at a TSS upstream of the protein-coding gene [102]. Interestingly, it was recently shown that the DNA repair gene *DEF1* is attenuated by Sen1 and CPA factors, without Nrd1 and Nab3 involvement [106]; therefore, PTT in *S. cerevisiae* might not be limited to the NNS pathway.

There are no Nrd1/Nab3 homologues known in plants. However, PTT has an elaborate role in the control of flowering time in *Arabidopsis thaliana*. FLC is a transcription factor that acts as a master regulator of flowering. It is carefully titrated: small changes in *FLC* transcript levels significantly affect flowering. The accumulation of *FLC* mRNA is prevented by FCA and FPA, two RNA-binding proteins associated with RNA 3′-processing factors. FCA and FPA autoregulate their own levels by premature polyadenylation and termination, independently of each other 107., 108.. Interestingly, they also promote early termination of the lncRNA *COOLAIR* 108., 109.. *COOLAIR* is an antisense transcript to *FLC*, and functions in early cold-induced silencing of *FLC* transcription [110]. As a result, several layers of premature termination of coding and noncoding transcripts act to control the timing of plant flowering. It is likely that other examples of PTT will be established in plants.

Alt-text: Box 1

## TSS-Linked Premature Transcription Termination

PTT of a protein-coding gene can be divided into termination events occurring close to the TSS or within the gene body (Figure 1). We predict that PTTs at these two locations are likely to be functionally and mechanistically different, since they occur at different stages of the transcription cycle.

For many genes, their TSS is characterised by high accumulation of Pol II, as measured by chromatin immunoprecipitation (ChIP). This depends on the action of negative elongation factor (NELF) and DRB sensitivity-inducing factor (DSIF), typically occurring 30–50 base pairs downstream of the TSS [17]. Such a Pol II ‘pileup’ is usually interpreted as stable pausing of engaged Pol II. However, it may be also due to PTT with concomitant Pol II turnover [18]. Several lines of evidence support the latter: termination and RNA 3′-processing factors have been observed to accumulate at the 5′ ends of genes 19., 20., short nuclear capped transcripts have been detected [21], and RNA cleavage sites near the TSS were identified [22]. Over the past 2 years, more direct experiments have demonstrated that a high percentage of TSS-bound Pol II molecules terminate prematurely.

Pol II binding to the genome was recently measured at a single-molecule resolution in *Drosophila*, with the aid of a novel single-molecule footprinting method [23]. This revealed unexpectedly high levels of Pol II turnover at the promoters of paused genes. In particular, the measured Pol II half-life at promoters of model paused genes was comparable to ‘nonpaused’, normally elongating genes. Therefore, Pol II accumulation at these promoters appears to be largely due to PTT, rather than to stable pausing of transcription-competent polymerases. This interpretation is further supported by an independent study that analysed the real-time dynamics of Pol II in live human cells using fluorescence recovery after photobleaching (FRAP) [24]. Computational modelling of Pol II kinetics showed that initiating Pol II remains chromatin bound for only 2.4 s and promoter-paused Pol II for 42 s, in contrast to elongating Pol II, which remained chromatin bound on average for 23 min. These big differences in Pol II residence times suggest that only a small fraction of initiating and pausing Pol II proceeds through a complete transcription cycle, whereas most Pol II is released from chromatin at the promoter. Indeed, the determined rate constants showed that only ~10% of Pol II molecules that initiate transcription will go on to promoter pausing and, of those, only ~10% continue into productive elongation. Thus, this study indicates that 99% of transcription initiation events result in PTT at the promoter, with only 1% giving rise to mRNA [24]. This surprisingly inefficient transcription initiation process is consistent with previous Pol II measurements on a *lac*O array [25]. Furthermore, the inhibition of PTT is the most plausible explanation for the dramatic increase in promoter-associated Pol II within 2–3 min after H_2_O_2_ addition to U2OS cells [26]. As a further clever way to investigate promoter-associated Pol II, the differential sensitivity of transcription initiation and elongation to high ionic strength has been used [27]. This showed that blocking recruitment of Pol II to promoters (but not elongation) by high salt treatment affected its binding in **ChIP followed by next-generation sequencing** (ChIP-seq), and revealed an almost complete loss of Pol II from promoter-proximal pause sites within 2–5 min. This loss was rapidly reversible and unaffected by transcriptional inhibitors. Therefore, Pol II removal from pause sites appears not to require elongation. Instead, a high rate of assembly and eviction of pre-initiated Pol II complexes at TSS is predicted. Although the above-mentioned studies used different methodologies, they all describe high turnover rates of Pol II at *Drosophila* or human promoters in various cell types 23., 24., 25., 26., 27.. Therefore, it is unlikely that the observed turnover is an artefact from any one procedure or an unusual cell type. In conclusion, most initiating Pol II molecules appear to terminate prematurely. It follows that the release of Pol II into productive elongation may be regulated by inhibition of this promoter proximal Pol II termination.

While promoter-proximal PTT has been largely overlooked, Pol II pausing in this location is well established in metazoans, and is tightly regulated by negative and positive elongation factors, such as P-TEFb 17., 28., 29.. Several previous studies described longer median half-lives of paused Pol II 30., 31., 32., 33.. One possible explanation for this discrepancy is the use of triptolide to block initiation. This blocks open complex formation by inhibition of TFIIH-associated XPB 34., 35. and was assumed to prevent recruitment of stable Pol II complexes at promoters. However, triptolide also disturbs transcriptional regulation and Pol II stability. Additionally, there is a lag in the onset of XBP inhibition, which may prevent accurate half-life determination 24., 27.. Further studies using different drugs and methods are required to resolve this discrepancy and so determine the relative contribution of promoter-proximal Pol II pausing versus PTT, to Pol II occupancy at promoters and to the control of productive elongation. Although technically challenging, it will be important to more directly determine the percentage of RNA molecules associated with paused Pol II, which, in physiological conditions, lead to mRNA production; that is, to demonstrate their assumed precursor–product relationship. Notably, paused Pol II blocks transcription initiation of additional polymerases 33., 36.. As Pol II residence times are variable on different genes 23., 33. the relevance of PTT and Pol II pausing is likely to be gene specific. Importantly, the two models explaining promoter-associated Pol II accumulation are not mutually exclusive; Pol II pausing may even have a direct role in early termination. Currently the mechanism of TSS-associated PTT remains unclear. For example, does Pol II disengagement from DNA in the vicinity of the TSS require a prior RNA cleavage step?

## Intragenic Premature Termination of Transcription

Once Pol II overcomes the TSS-associated elongation checkpoint, subsequent PTT likely requires a co-transcriptional cleavage reaction at a cryptic **polyadenylation site** (PAS), mainly located in introns. PAS-mediated cleavage within internal exons appears to be a small fraction compared with introns, and leads to transcripts without a stop codon, which typically get rapidly degraded through the nonstop decay pathway [37], although, in rare cases, they can result in truncated proteins [38]. PTT in the beginning of the first intron is frequent in conditions were **U1 small nuclear ribonucleoprotein complex (U1 snRNP)** or the **cap-binding complex** (CBC) are depleted 39., 40., 41., and partially coincides with a second Pol II stalling event at stable nucleosomes downstream of CpG island promoters [42]. Such PTT leads to unstable transcripts that undergo rapid degradation (see next section). Most current knowledge of intragenic PTT comes from the analysis of stable premature polyadenylated transcripts, which can be readily detected by various RNA-sequencing methodologies. These stable transcripts are classified as instances of APA, as reviewed in [14]. APA within the gene body, upstream of the 3′-UTR, has been referred to as coding sequence APA (CDS-APA), upstream region APA (UR-APA), **intronic polyadenylation** (IPA/IpA) or alternative last exon (ALE). We use the term ‘IPA’ here. IPA was first described for specific genes in 1980 43., 44., 45., but only recently has its widespread nature and significance been appreciated, due to the development of different PAS-sequencing methods. Thus, ~40% of murine genes [46] and 16% of genes in human immune cells [47] have been reported to express IPA isoforms. In particular, IPA has been shown to be a frequent genome-wide event, with diverse roles in immune cells [48], inactivation of tumour suppressor genes [49] ,and regulation of DNA repair genes [50]. Notably, a systematic analysis of IPA in normal human tissues, primary immune cells, and multiple myeloma samples has been used to create an atlas of 4927 high-confidence IPA events in these cell types [48]. IPA isoforms were shown to yield stable transcripts, which, in the case of 5′-proximal IPA, tend to produce **noncoding RNA** (ncRNA). By contrast, more 3′-proximal IPA events tend to produce truncated proteins [48].

## Fate and Function of Prematurely Terminated Transcripts

**Figure 2:**
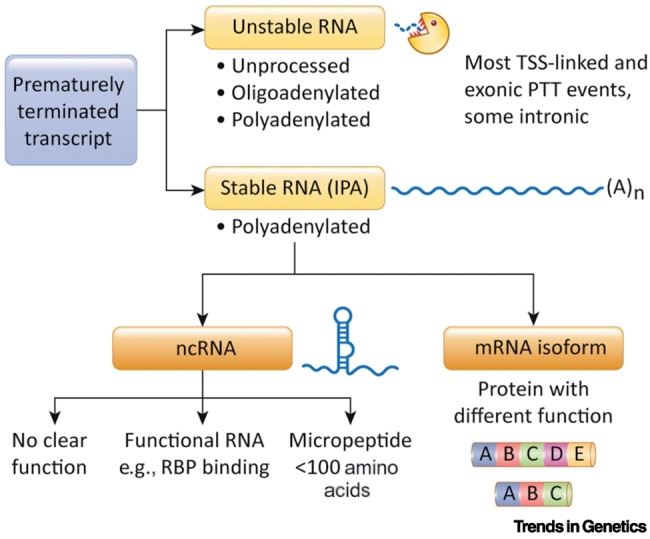
Fate and Function of Prematurely Terminated Transcripts. Most transcription start site (TSS)-linked, exonic, and some intronic prematurely terminated transcripts are unstable, and likely lack cellular function. Stable prematurely terminated transcripts generated as a result of intronic polyadenylation (IPA) form either a noncoding (nc) RNA or protein-coding mRNA. Contrary to their name, ncRNA sometimes contain small open reading frames (ORFs), which may be translated into micropeptides. Other ncRNA can serve cellular functions, for example as scaffolds for RNA-binding proteins (RBP) [48]. Many ncRNA have no clear function determined to date. Protein-coding mRNA isoforms generated by PTT diversify the proteome. They lack the C-terminal domain(s) present in the full-length protein and may have different properties. These include membrane binding versus soluble 43., 44., 45., 48., 57., altered specificity or affinity for binding to nucleic acid or protein partners [48], and, in some cases, dominant negative functions 59., 60., 61., 62., 63.. Abbreviation: (A)_n_, polyadenylation.

PTT may lead to three outcomes: rapid transcript degradation; formation of a more stable ncRNA; or production of a protein-coding, polyadenylated mRNA isoform (Figure 2). The more 5′ proximal a transcript is terminated, the more likely it is to be degraded. TSS-linked PTT might not require cleavage and could lead to unprocessed, unprotected, nonpolyadenylated RNA. Further downstream, transcripts may be cleaved and at least partially polyadenylated. Short transcripts appear to generally undergo quick degradation, similar to **promoter upstream transcripts** (PROMPT)/upstream antisense RNA, which also often yield **exosome**-sensitive RNA 22., 51., 52., 53.. In the case of short sense transcripts, while PAS usage allows for transcript cleavage, this might not lead to efficient polyadenylation. Another explanation for the instability of short transcripts is that the 3′ entry site for the nuclear exosome in such transcripts is physically close to the 5′-capped end of the transcript, which is bound by the CBC, known to contribute to exosomal decay, especially of some classes of ncRNA 41., 54., 55.. Overall, different surveillance pathways may operate at different parts of the gene. It is also plausible that productive 3′-end processing and RNA stability are transcript dependent and influenced by the new coding potential of the shorter isoform, its new 3′-UTR regulatory elements, and so on.

Stable prematurely terminated RNAs are mainly polyadenylated and contribute to the IPA transcriptome. They can be further subdivided into ncRNA and protein-coding mRNA isoforms (Figure 2). Some ncRNA contain an **open reading frame** (ORF) and, therefore, might produce a micropeptide (<100 amino acids). Other ncRNA could serve specific functions. For example, PTT of *ASCC3* leads to the formation of a stable ncRNA, which is critical for the recovery of transcription following ultraviolet (UV) damage [56]. IPA-generated ncRNA also interact with RNA-binding proteins, which are normally enriched in the 3′-UTRs of coding transcripts, such as FUS, ELAVL1, PUM2, TAF15, and TIAL1 [48]. Such ncRNAs could act as scaffolds for RBPs and so regulate other RNA in *trans*.

In the third scenario, protein-coding IPA isoforms (Figure 2) contribute to the diversity of the proteome, for example by generating proteins with physiologically distinct functions, or truncated dominant negative proteins. In the classic case of B cell expressed immunoglobulin M heavy chain mRNA, cellular activation causes a switch from full-length to IPA mRNA isoforms, resulting in a change from membrane-bound to secreted forms of the antibody 43., 44., 45.. IPA appears to regulate membrane-anchoring properties of many other proteins. Computational analysis revealed that 376 mouse genes are likely to use the IPA mechanism to generate proteins with changes to their transmembrane domains (TMD) [57]. In human cells, although TMD-containing proteins are significantly depleted among IPA genes, 499 genes encoding transmembrane proteins undergo IPA and, in 152 cases, these lead to loss of the TMD [48]. For example, IPA generates various mRNA isoforms of the transmembrane T cell co-stimulator CD46, which are predicted to form soluble CD46 [58]. Similarly, PTT variants encoding proteins with dominant negative functions, such as retinoblastoma-binding protein 6 (RBBP6) 59., 60., MAGI3 61., 62. or platelet-derived growth factor receptor α (PDGFRα), have been also described [63]. Interestingly, the site of premature polyadenylation is frequently located within a domain mediating either protein–protein interactions or DNA or RNA binding, such as zinc finger arrays [48]. The partial loss of such interaction surfaces may lead to altered binding affinities for protein interaction partners and altered nucleic acid-binding specificity, respectively. Therefore, IPA may be physiologically relevant by allowing a diversification of protein function. By contrast, the widespread pathological use of premature polyadenylation has been uncovered in chronic lymphocytic leukaemia [49]. Here, mRNA truncations by IPA are recurrent and predominantly affect genes with tumour-suppressive functions. This leads to either their inactivation or transformation into oncogenes. In conclusion, proteome alteration occurring as a result of IPA can be either beneficial or harmful.

## Gene Regulation by Premature Transcription Termination

**Figure 3:**
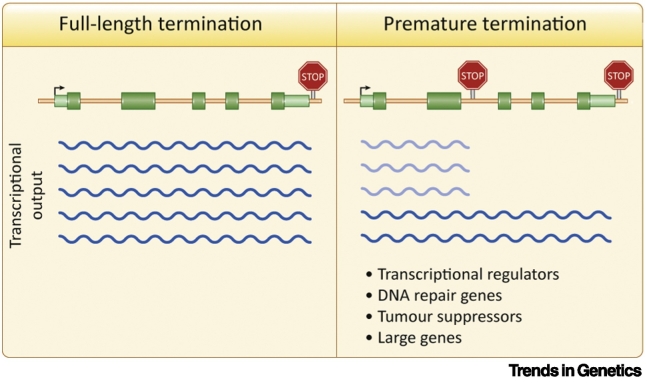
Transcriptional Control by Premature Termination. Premature transcription termination (PTT) is mutually exclusive with full-length transcription. Independent of the fate or function of prematurely terminated transcripts, PTT negatively regulates the expression of the full-length transcripts. Genes controlled by PTT include transcriptional regulators 48., 68., DNA repair genes [50], and tumour suppressor genes [49], and tend to be larger than average genes 40., 69..

Regardless of whether the prematurely terminated transcript is stable or unstable, and independently of its ability to produce a functional ncRNA or protein, a potential outcome of early termination is the repression of the corresponding full-length mRNA (Figure 3). Such negative regulation has been demonstrated for the *CSTF3* gene (known also as *CSTF77*), originally in *Drosophila* and then in humans 64., 65., 66.. CSTF3 is a CPA complex subunit, stimulating cleavage. Premature CPA of *CSTF3* is induced by high levels of CSTF3 protein, thus forming a negative feedback loop controlling its own activity, which is important for cell cycle control [66]. The CSTF3 example suggested that PTT could be a more general mechanism to repress gene expression in metazoans.

Supporting this possibility, the abundant U1 snRNP has been shown to function not only in splicing, but also by blocking widespread PTT in thousands of vertebrate genes 39., 40.. Vertebrate genes often contain large introns, with numerous cryptic **poly(A) signals**. Therefore, they are inherently susceptible to PTT, although this is suppressed by U1. Interestingly, PTT is strongly activated upon UV treatment of cells, and correlates with decreased U1 snRNA levels [67] and a slowdown in transcription elongation [56]. These studies demonstrated the widespread predisposition of vertebrate genes to IPA. However, it was unclear whether PTT serves a regulatory role genome wide, or is a harmful genomic accident, induced by DNA damage and suppressed by U1. Physiological regulation is usually achieved by limiting factors, whereas U1 snRNP is extremely abundant, at ~1 million copies per human cell.

Finally, it was recently shown that a subset of protein-coding genes is downregulated by PTT under physiological conditions, both in cultured human cells and during zebrafish embryogenesis. This is triggered by an RNA 3′-processing and termination factor called PCF11 [68]. Similar to CSTF3, PCF11 uses PTT as an autoregulatory mechanism 68., 69.. However, 218 other human genes were additionally identified as downregulated by PCF11-mediated PTT. This is likely an underestimate, because many genes undergoing PCF11-mediated PTT might also be dependent on PCF11 for efficient full-length transcript expression. Interestingly, half of the PCF11-attenuated genes show PTT without detectable IPA products [68]. This suggests that many transcripts generated by PTT under physiological conditions are unstable and that IPA corresponds to only a subset of intragenic PTT. Therefore, it will be important that future PTT studies also assay the nascent transcriptome. Notably, genes undergoing PCF11-mediated PTT are enriched for transcriptional regulators, both in humans and in zebrafish [68]. Similarly, in primary human tissues, IPA has been shown to occur preferentially on genes encoding transcriptional regulators [48]. PCF11 levels are an order of magnitude lower compared with other 3′-processing factors, but vary between tissues, making PCF11 a likely regulatory factor [68]. Overall, those data indicate that PTT is a naturally occurring, widespread, and controlled phenomenon in vertebrates.

## The Role of CPA and Termination Factors in PTT

**Figure 4:**
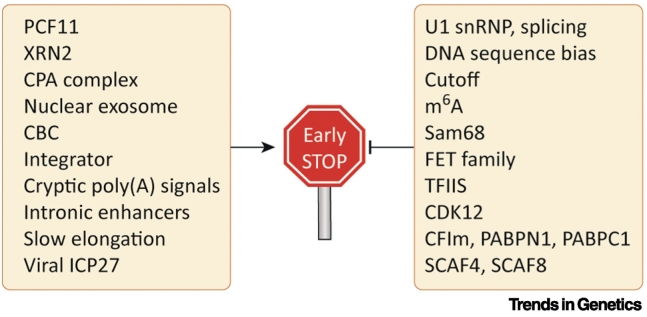
Factors Triggering and Suppressing Premature Transcription Termination (PTT). The molecular mechanism of PTT is unclear. However, multiple factors may trigger or prevent PTT. PTT-triggering factors include not only canonical proteins involved in RNA 3′-processing and transcription termination at gene ends [PCF11, XRN2, and the cleavage and polyadenylation (CPA) complex], but also other factors, mainly involved in RNA processing [nuclear exosome, cap-binding complex (CBC), and the Integrator complex]. Intragenic PTT is mutually exclusive with splicing; therefore, the process of splicing and splicing-promoting factors are major factors suppressing PTT. Further players preventing spurious PTT are also shown, and discussed in the main text. We note that many of the listed factors affect PTT indirectly, by affecting RNA polymerase II (Pol II) dynamics, co-transcriptional processing, or RNA stability. Further mechanistic studies are needed to provide proof of which factors have a direct role in PTT. Abbreviations: CFIm, mammalian cleavage factor I; m^6^A, modification of RNA by *N*6-methyladenosine.

Since PCF11 acts at both gene ends and throughout the gene body, it appears likely that other canonical CPA and termination factors are involved in PTT (Figure 4). Notably, XRN2, the nuclear 5′–3′ exonuclease ‘torpedo’ that facilitates transcription termination at the 3′ ends of genes, has been shown by ChIP-seq to also localise near TSS, and interact with **decapping** factors [19]. Coupled decapping of nascent transcripts and PTT has been suggested to limit bidirectional Pol II elongation. PCF11 as well as CPSF73 is enriched at TSS in ChIP-seq [68]. Additionally, *in vivo* crosslinking and immunoprecipitation (CLIP) revealed that all analysed CPA factors (CPSF73, CstF64, CstF64t, CPSF160, CPSF30, and CFIm25) were significantly detected on both strands in the vicinity of the TSS 70., 71.. Finally, depletion of CPA and termination factors (CPSF73, CSTF64, XRN2, and PCF11) resulted in increased **mammalian native elongating transcript sequencing (mNET-seq)** signal specifically at the TSS in both sense and antisense direction 68., 71.. This suggests that TSS-linked nonproductive RNA synthesis may be terminated by the same factors that are responsible for 3′ processing and transcription termination at gene ends.

Apart from PCF11, the involvement of the termination machinery in premature cleavage within the gene body is less well defined. However, IPA globally correlates with the preferential use of proximal PASs in 3′-UTRs 46., 48., 72., 73., which are both more prevalent in proliferating cells but less so during cell differentiation. Consequently, IPA and 3′-UTR APA are likely to be coregulated. In addition to PCF11, FIP1 is a likely candidate for PTT stimulation, because it is the only other CPA factor known to promote early PAS usage [74].

## Noncanonical Factors Involved in Premature Termination

Besides the factors that are known to mediate 3′-processing and transcription termination at gene ends, others have emerged that stimulate PTT (Figure 4). In budding yeast, premature termination is tightly linked with RNA degradation by the nuclear exosome [55]. Similarly, mutation of the catalytic subunit of the human nuclear exosome complex, DIS3, resulted in accumulation of truncated RNA, likely to be PTT products [75]. Furthermore, the mammalian nuclear exosome interacts with the CBC. Two CBC-associated proteins, ZC3H18 and SRRT/2, have also been connected with PTT events in the first introns of protein-coding genes [41].

Another candidate factor for PTT is the Integrator complex. This multiprotein complex interacts with Pol II and was initially identified as a RNA 3′-processing complex for snRNA 76., 77.. Two Integrator subunits, IntS9 and IntS11, are homologous to the CPA factors CPSF100 and CPSF73, respectively [78]. More recently, experimental evidence emerged that suggested that Integrator also has a role in the activation of protein-coding genes, particularly in Pol II pause–release and elongation 79., 80., 81., 82.. The subunit containing endonuclease activity, Ints11, was found to bind around TSS and to be required for TSS–proximal Pol II pause–release [80]. This function was dependent on its endonucleolytic activity. Thus, it is conceivable that the Integrator complex is involved in TSS-associated PTT.

In terms of the genetic elements that promote PTT, cryptic poly(A) signals within introns are likely candidates. Interestingly, intragenic enhancers also appear to stimulate PTT, because transcription at these enhancers interferes with, and attenuates, host gene transcription during productive elongation [83]. Further genetic elements might also exist that can trigger PTT.

## Factors Opposing Premature Termination

Since PTT is disruptive to full-length transcription, many mechanisms and factors must exist to suppress this process (Figure 4). A major factor in preventing PTT is U1 snRNP, as described earlier. Furthermore, DNA sequences likely evolved to prevent harmful, yet reinforce beneficial, termination events. Around the TSS, poly(A) signals are depleted in the sense direction relative to the upstream antisense direction, while U1 snRNP recognition sites show the opposite pattern. This sequence asymmetry has been proposed to control promoter directionality 22., 53.. It has also been reported that codon usage biases coevolve with transcription termination machinery to suppress PTT and allow optimal gene expression [84]. Termination inhibition can also occur in a chromatin-dependent, and sequence-independent manner, as shown in *Drosophila* for the piwi-interacting RNA (piRNA) transcription regulator Cutoff. Cutoff prevents cleavage of nascent RNA at PAS by interfering with recruitment of the CPA complex and also protects processed transcripts from degradation [85]. In addition, RNA modifications might prevent PTT, because a negative correlation between N6-methyladenosine (m^6^A) modification of mRNA and PTT has been reported [62]. Other factors that could protect against PTT are RNA-binding proteins. For example, in neural progenitor cells, Sam68 binding to an intronic PAS in *Aldh1a3* prevented its recognition and consequent PTT, promoting cell self-renewal [86]. It was further demonstrated in male germ cells that Sam68 interacts with U1 snRNP and is required for U1 snRNP recruitment to Sam68-regulated intronic PAS [87]. In addition, the FET family of proteins (FUS, EWSR1, and TAF15), mutations in which cause amyotrophic lateral sclerosis, has been demonstrated to interact with U1 snRNP 88., 89. and might participate in U1 snRNP-mediated PTT prevention.

Modifying Pol II activity may be another way to oppose PTT. When Pol II misincorporates a base or runs into a ‘roadblock’ such as a nucleosome, it arrests and backtracks. Pol II elongation is rescued by TFIIS stimulating the RNA endonuclease activity of Pol II, which produces a new 3′ end in the active site. It was recently shown that TFIIS stimulation of Pol II cleavage activity antagonises both premature TSS-proximal and gene end termination [90]. Pol II is further regulated by phosphorylation of the CTD of its largest subunit. Depletion of the CTD kinase CDK12 results in genome-wide increased IPA, indicating that CDK12 suppresses PTT [50]. Interestingly, many DNA repair genes harbour more IPA sites than other expressed genes, and are particularly sensitive to the loss of CDK12. IPA is also opposed by CPA factors that favour distal APA events, especially the CFIm complex subunits, PABPN1 and PABPC1 74., 91.. While this article was in review, a new study described a role for previously uncharacterised Pol II CTD-binding proteins SCAF4 and SCAF8 in binding to RNA upstream of early PAS and suppressing PTT in ~1300 human genes. Interestingly, both factors interact with the CPSF subcomplex of the CPA machinery; therefore, the mechanism of SCAF4 and SCAF8-mediated PTT prevention might involve an effect on the CPA complex [92].

Since premature cleavage and polyadenylation are mutually exclusive with splicing of the intron in which the PAS is located, competition between splicing and PTT can be anticipated. Indeed, knockdown of various splicing factors as well as inhibition of 5′ splice site recognition by antisense nucleotide consistently results in IPA 49., 74.. Therefore, antagonism between splicing and PTT goes beyond U1 snRNP-mediated PAS blocking, as described earlier. Further to splicing, Pol II elongation rates have been shown to influence PTT, because IPA events increase under conditions where Pol II elongation is slowed 93., 94., 95.. Slower Pol II provides a longer window of opportunity for CPA before the 3′ splice site is reached. Hence, changes in the balance between splicing and PTT, together with Pol II pausing, predispose it to termination.

## Concluding Remarks and Future Perspectives

Here, we have outlined recent evidence that demonstrates the widespread occurrence of PTT in protein-coding genes, both TSS proximal and intragenic. PTT limits and opposes full-length transcription. Consequently, it may contribute to pathological processes, such as host gene downregulation during viral infection [96] and to carcinogenesis by inactivation of tumour suppressor genes [49]. PTT is globally suppressed by genic DNA sequence biases, and various cellular factors, such as U1 snRNP. By contrast, PTT can be also beneficial: it diversifies the transcriptome and proteome, and contributes to gene regulation. Gene regulation by PTT occurs in at least four kingdoms of life: Eubacteria, Fungi, Plantae, and Animalia (Box 1). Yet, the regulatory aspect of PTT has been largely overlooked in metazoan research. This is partially because the machinery triggering PTT varies in different organisms. The well-researched Nrd1–Nab3–Sen1 (NNS) complex regulating PTT in *Saccharomyces cerevisiae* is absent not only in plants and animals, but also in the less evolutionarily distant *Schizosaccharomyces* *pombe*. Even so, the mechanisms modulating PTT appear conserved. In budding yeast, NNS cooperates with PCF11 [97], and both NNS and PCF11 levels are controlled by PTT 98., 99.. In vertebrates, PCF11 uses PTT to regulate own levels, as well as the levels of other 3′-processing factors and transcriptional regulators [68]. Therefore, vertebrate PCF11 may have at least partially coopted the function of the yeast NNS complex. Currently, many questions about the regulation and function of PTT remain (see Outstanding Questions). In particular, we do not understand the mechanisms that lead to, or prevent, PTT. It is also unclear which of the factors enhancing and preventing PTT are able to do so directly. Despite these current gaps in our knowledge, we anticipate that PTT in metazoans is a critical feature of gene regulation. It is also possible that this process can be manipulated to achieve clinical and biotechnological benefits (Box 2).

Box 2 **Implications of PTT in Medicine and Biotechnology**

Aberrant PTT is associated with several pathological processes: inactivation of tumour suppressor genes in chronic lymphocytic leukaemia [49]; downregulation of the expression of Kv11.1 potassium channel leading to a heart rhythm condition [111]; and loss of neuronal growth-associated factor stathmin-2 in neurodegeneration [112]. Blocking pathological PTT by antisense morpholinos has been suggested as a potential therapeutic strategy [113]. Conversely, induction of PTT to prevent formation of disease-inducing transcripts has also been proposed as a therapeutic approach. In particular, the insertion of intronic poly(A) signals upstream of toxic expanded CTG repeats in the *DMPK* gene as associated with myotonic dystrophy type 1 was demonstrated to revert the pathological phenotype of patient-derived induced pluripotent stem cells [114]. Similar approaches could also be used in biotechnology. Of note, two small-molecule modulators of APA have been discovered that promote distal-to-proximal APA usage [115]. These could prove a useful tool for APA and PTT manipulations.

Alt-text: Box 2

## Outstanding Questions

What is the relative contribution of Pol II pausing and transcription termination to Pol II accumulation at promoters and to the control of transcription elongation?
What is the molecular mechanism of PTT and which pathways are involved? Is PTT always preceded by RNA cleavage?
What is the role of canonical RNA 3′-processing and termination factors in PTT? Are early and normal termination processes at gene ends co-regulated?
How are the prematurely terminated RNAs processed and what determines their stability?
Which biological processes depend on early termination? Which PTT events are beneficial and which are harmful? How widespread is transcriptional regulation by PTT in physiological conditions?
In *Saccharomyces cerevisiae*, many prematurely terminated transcripts use different TSS to the full-length transcript. Does this also occur in metazoans?
Which factors control PTT directly and which influence it indirectly? What further factors trigger or prevent PTT?

## Glossary

**Alternative polyadenylation (APA)**: generation of alternative transcripts differing at their 3′ ends, occurring through usage of multiple poly(A) signals in one gene.
**Cap-binding complex (CBC)**: a protein complex binding the 5′ cap structure of eukaryotic mRNA and protecting it from decapping and degradation.
**Chromatin immunoprecipitation followed by next-generation sequencing (ChIP-seq)**: a method to assess the genome wide localisation of a protein on chromatin.
**Cleavage and polyadenylation (CPA)**: RNA-processing events occurring at the 3′ ends of pre-mRNA, leading to the formation of a mature mRNA.
**Decapping**: removal of the 5′ cap structure from RNA, predisposing it to degradation.
**Exosome**: a multisubunit protein complex harbouring 3′−5′ exoribonucleolytic and endoribonucleolytic activities that functions in RNA degradation.
**Intronic polyadenylation (IPA)**: APA occurring within an intron of a protein-coding gene, leading to a truncated transcript; also known as coding sequence APA (CDS-APA), upstream region APA (UR-APA), or alternative last exon (ALE); a subtype of PTT.
**Mammalian native elongating transcript sequencing (mNET-seq)**: a technique that maps nascent transcripts associated with specific Pol II isoforms in mammalian cells.
**Noncoding RNA (ncRNA)**: nonprotein-coding transcripts (note that some ncRNA might encode small peptides).
**Open reading frame (ORF)**: part of a transcript that can be translated, comprising a continuous stretch of codons beginning with a start codon and ending with a stop codon.
**Polyadenylation signal [Poly(A) signal]**: signal in the nascent RNA (typically including the hexamer AAUAAA) promoting RNA cleavage and polyadenylation.
**Polyadenylation site (PAS)**: RNA cleavage site to which poly(A) is added, typically located 10–30 nucleotides downstream of the poly(A) signal.
**Premature transcription termination (PTT)**: early release of Pol II from the gene template between the TSS and 3′-UTR of the gene; sometimes also referred to as transcription attenuation.
**Promoter upstream transcripts (PROMPT)**: known also as upstream antisense RNA, a class of human transcripts that are produced upstream of the promoters of active genes in a divergent orientation to the main (coding) transcript.
**RNA Polymerase II (Pol II)**: enzyme responsible for transcribing all protein-coding mRNA in eukaryotic cells, along with many ncRNA.
**Transcription start site (TSS)**: the first nucleotide in the DNA sequence that undergoes transcription into RNA.
**Transcription termination**: the process whereby an RNA polymerase stops transcribing and dissociates from its DNA template.
**U1 small nuclear ribonucleoprotein complex (U1 snRNP)**: comprises the small nuclear RNA (snRNA) U1 and its associated proteins; crucial for splicing.
**Untranslated region (UTR)**: 5′ and 3′ parts of mRNA surrounding the ORF that are not translated into protein and often serve regulatory functions.
